# Norovirus Detection and Genotyping for Children with Gastroenteritis, Brazil

**DOI:** 10.3201/eid1308.070300

**Published:** 2007-08

**Authors:** Caroline C. Soares, Norma Santos, Rachel Suzanne Beard, Maria Carolina M. Albuquerque, Adriana G. Maranhão, Ludmila N. Rocha, Maria Liz Ramírez, Stephan S. Monroe, Roger I. Glass, Jon Gentsch

**Affiliations:** * Universidade Federal do Rio de Janeiro, Rio de Janeiro, Brazil; † Centers for Disease Control and Prevention, Atlanta, Georgia, USA; 1 Current affiliation: Fiocruz–Oswaldo Cruz Institute, Rio de Janeiro, Brazil

**Keywords:** Norovirus, viral gastroenteritis, viral diagnostics, real-time RT-PCR, dispatch

## Abstract

During 1998–2005, we analyzed stool samples from 289 children in Rio de Janeiro to detect and genotype norovirus strains. Previous tests showed all samples to be negative for rotavirus and adenovirus. Of 42 (14.5%) norovirus-positive specimens, 20 (47.6%) were identified as genogroup GI and 22 (52.3%) as GII.

Noroviruses, a genus within the family *Caliciviridae*, have emerged as an important cause of epidemic and sporadic diarrheal disease in humans of all ages worldwide (*1**–**3*). The norovirus genome consists of a single strand of positive-sense RNA organized into 3 open reading frames (ORFs). ORF1 encodes nonstructural proteins such as RNA-dependent RNA polymerase, ORF2 encodes viral capsid protein 1, and ORF3 encodes a small capsid protein (viral capsid protein 2) associated with stability of viral capsid protein 1 (*4*–*6*). According to nucleotide sequence analysis of the polymerase and capsid regions, noroviruses are classified into 5 genogroups, GI to GV; each genogroup can be further divided into several clusters or genotypes. Genogroups GI, GII, and GIV have been found in humans, though GII seems to be the predominant strain around the world (*4**,**7**–**11*). To detect and genotype norovirus in stool samples from Brazilian children <10 years of age who had acute diarrhea, we used real-time Light Cycler reverse transcription–PCR (RT-PCR) and conventional RT-PCR assays.

## The Study

From January 1998 through May 2005, a total of 2,421 fecal specimens were collected from children <10 years of age (median age 2.3 years) with acute diarrhea in Rio de Janeiro, Brazil. Of these, 478 (19.7%) specimens were collected from hospitalized children (inpatients) and 1,943 from outpatient children (341 [14.1%] from the emergency department and 1,602 [66.2%] from the walk-in clinic). Of these samples, 14.3% were positive for rotavirus (9.8%) or adenovirus (4.5%). The median age was 12.5 months for rotavirus-positive patients and 12.2 months for adenovirus-positive patients. Overall, of the hospitalized, emergency department, and walk-in clinic patients, 11.7%, 6.2%, and 10.0%, respectively, had samples positive for rotavirus, and 4.8%, 4.1%, and 4.4%, respectively, had samples positive for adenovirus. Enteropathogenic bacteria such as *Escherichia coli*, *Salmonella* spp., *Yersinia enterocolitica*, *Campylobacter* spp., and *Shigella* spp. were found in 8% of the specimens. Seven mixed infections were detected (2 adenovirus and *Salmonella* spp., 2 adenovirus and *E. coli*, 1 adenovirus and *Campylobacter* spp., 1 rotavirus and *Salmonella* spp., and 1 rotavirus and *E. coli*).

We selected 289 specimens that represented a random subset of samples that had prior negative results for rotavirus and enteric adenovirus. Of these 289, 117 were collected from inpatients and 172 from outpatients (89 emergency department and 83 walk-in clinic). The mean and the median age of the tested patients was 3.1 years. Suspensions of stool (10%) were prepared in diethylpyrocarbonate-treated water and Vertrel XF (Miller-Stephenson, Sylmar, CA, USA) and clarified by centrifugation at 2,100× *g* for 10 min. We used 200 μL of suspension for RNA extraction with the NucliSens extraction kit (Organon Tekninka, Durham, NC, USA) according to the manufacturer’s instructions. The RNA was eluted in 50 μL of elution buffer and stored at –70°C until use.

A total of 240 samples were tested for norovirus RNA by Light Cycler PCR that used primers and probes for ORF1/ORF2 junction region specific for norovirus GI and GII, as described (3*,**12*), and by the Light Cycler RNA Amplification Kit Hybridization Probes (Roche, Basel, Switzerland). Samples that showed a positive threshold at <38 cycles were considered positive. The 49 remaining samples were tested only by conventional RT-PCR, as described (*5*). Conventional RT-PCR was performed with the QIAGEN OneStep RT-PCR Kit (QIAGEN, Valencia, CA, USA). The RNA samples were subjected to 1 cycle of reverse transcription (42°C, 10 min) followed by 5 min at 95°C. PCR was performed for 40 cycles, each consisting of 1 min at 94°C, 1 min at 40°C (for GI detection) or 1 min at 44°C for (for GII detection), 1 min at 72°C, and a final extension cycle of 10 min at 72°C.

We selected 6 samples that were positive by real-time Light Cycler PCR (2 GI and 4 GII) for analysis by conventional RT-PCR with specific primers in capsid region D of norovirus GI and GII, as described above. The amplified cDNA samples were purified from the gel by using QIAquick gel extraction kit (QIAGEN), and the sequences were determined with the BigDye terminator cycle sequencing kit and the ABI PRISM 3100 automated DNA sequencer (Applied Biosystems, Foster City, CA, USA) by using the same primers as used for the conventional RT-PCR. The nucleotide sequences of the amplicons were aligned with corresponding sequences of selected norovirus strains available in the GenBank database and analyzed by using the CLUSTAL V algorithm of the MegAlign program in the DNASTAR software package (Madison, WI, USA). The nucleotide sequences obtained in this study were deposited in GenBank under accession nos. DQ496212, DQ496213, DQ496214, DQ496215, DQ496216, and DQ496217.

Of the 289 fecal specimens tested, 42 (14.5%) were positive for norovirus: 36 (15%; n = 240) by real time Light Cycler PCR and 6 (12.2%; n = 49) by conventional RT-PCR. These percentages correspond only to single infections because we did not test samples already known to be positive for other pathogens such as rotavirus and adenovirus. Positive samples and genogroups varied by year with no obvious yearly pattern (Table 1). Although norovirus is often referred to as “the winter vomiting disease,” we detected infection throughout the year, with no seasonal pattern (Figure). Norovirus infections were equally common among outpatients and inpatients. Among 117 inpatients, 18 (15.4%) had positive norovirus test results compared with 24 (14%) of 172 outpatients (11 emergency department and 13 walk-in clinic). Although the disease caused by norovirus is described as mild (diarrhea, vomiting, abdominal pain, and fever) and generally does not lead to hospitalization (*13*,*14*), of 42 norovirus-infected children, 29 (69%) were either hospitalized or received medical care in the emergency department, which suggests that they had a more severe illness. Only 13 (31%) of the 42 norovirus-infected children attended the walk-in clinic, which suggests that they had mild disease (Table 2). Other than diarrhea, fever was the most common symptom among the 42 norovirus-positive patients in this study, reported for 11 (26.2%) patients. Vomiting only was described for 8 (19.0%); vomiting and fever was described for 6 (14.3%). No mixed infection with bacteria was observed.

**Table 1:** Distribution of norovirus-positive samples detected in Rio de Janeiro, Brazil, 1998–2005*

Year	Real-time reverse transcription–PCR			Conventional reverse transcription–PCR		Total no. positive samples/genogroup
	No. samples tested	No. positive samples/genogroup		No. samples tested	No. positive samples/genogroup	
1998	23	4/3GI + 1GII		0	NA	4/3GI + 1GII
1999	29	3/GII		5	1/GI	4/1GI + 3GII
2000	31	4/2GI + 2GII		7	0	4/2GI + 2GII
2001	26	4/GII		0	NA	4/GII
2002	31	5/4GI + 1GII		10	0	5/4GI + 1GII
2003	32	8/5GI + 3GII		9	1/GI	9/6GI + 3GII
2004	39	4/1GI + 3GII		16	3/GI	7/4GI + 3GII
2005	29	4/GII		2	1/GII	5/5GII
Total	240	36/15GI + 21GII		49	6/5GI + 1GII	42/20GI + 22GII

*NA, not applicable.

**Figure:**
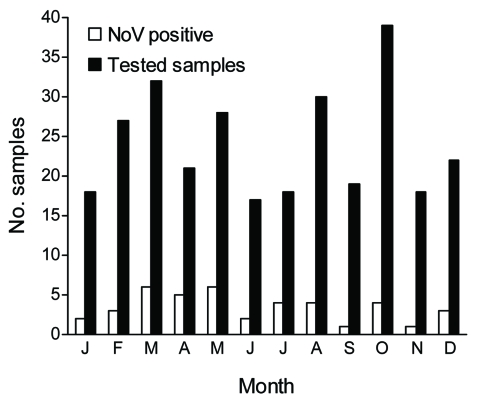
Seasonal distribution of norovirus (NoV) infections in Rio de Janeiro, Brazil, 1998–2005.

**Table 2:** Distribution of all tested samples by age groups and patient status, Rio de Janeiro, Brazil, 1998–2005

Age, y	Outpatients				Inpatients		
	No. tested	PCR-positive, no. (%)	PCR-negative, no. (%)		No. tested	PCR-positive, no. (%)	PCR-negative, no. (%)
<1	29	4 (13.8)	25 (86.2)		45	5 (11.1)	40 (88.9)
1–5	64	9 (14.0)	55 (86.0)		88	15 (17.0)	73 (83.0)
6–10	24	5 (20.8)	19 (79.2)		39	4 (10.3)	35 (89.7)

Although norovirus belonging to genogroup GII is considered the most prevalent strain worldwide (*7**–**9**,**11**,**15*), we found no important difference in the prevalence of the 2 genogroups detected in our study. Overall, 20 (48%) of the 42 samples were identified as genogroup GI and 22 (52%) as GII (Table 1). No statistically significant difference in the prevalence of GI and GII was observed between inpatients and outpatients.

## Conclusions

Our study documents that noroviruses are a common cause of acute gastroenteritis in children who are inpatients or outpatients in Brazil and are likely second only to rotavirus as a cause of severe childhood diarrhea. Our study was exploratory and has limitations. Nonetheless, it documents how common norovirus infections may be and indicates that further study will be necessary to assess their role among Brazilian children, to understand the epidemiology of the disease, and to seek evidence of immunity in children, which might encourage development of a vaccine.
